# Reflection-based questioning: Aspects affecting Myanmar students’ reading comprehension

**DOI:** 10.1016/j.heliyon.2022.e09864

**Published:** 2022-07-04

**Authors:** Tun Zaw Oo, Anita Habók

**Affiliations:** a Doctoral School of Education, University of Szeged, Hungary; b Institute of Education, University of Szeged, MTA-SZTE Digital Learning Technologies Research Group, Hungary

**Keywords:** Reflection-based questioning, Reading comprehension, Pre- and post-tests, Student questionnaire, Observation scheme

## Abstract

This study aimed to scrutinize the effects of the reflection-based questioning approach (RBQA) on Myanmar students’ achievement in English reading comprehension. The RBQA approach covers Oo et al.’s (2021) reflective teaching model for reading comprehension (based on *planning, acting, reflecting,* and *evaluating*) in which the teacher uses a questioning strategy (initiate-response-evaluate model). Employing cluster randomized trials, quasi-experimental research was conducted to investigate RBQA’s effectiveness in teaching reading comprehension skills to Grade-9 students. The experimental group (*N* = 228) received the RBQA intervention; the control group (*N* = 230) did not receive the intervention but was provided with traditional instruction. During RBQA intervention, teachers used the anonymous student questionnaire and observation scheme as effective reflection tools. After a five-week intervention, both groups completed post-tests to assess their achievement. The study findings revealed that teaching with RBQA had a significant positive effect on students’ reading comprehension. Therefore, this study is of immense significance to English language teachers and their students.

## 1 Introduction

In English language teaching (ELT), reading is emphasized as the most important skill among listening, speaking, writing, and reading (Rodli and Prastyo, 2017). Reading is also the most fundamental skill for nearly all academic subjects, students’ educational success, and their later careers (Okkinga et al., 2018). Therefore, in teaching reading comprehension skills, teachers should use the most effective teaching strategies to stimulate students’ critical thinking (Yuliawati et al., 2016). Among those strategies, the questioning strategy can affect students’ active learning participation (Nuryani et al., 2018), so most teachers currently use the questioning strategy to elicit students’ responses, check their understanding, and control their behavior (Yuliawati et al., 2016). Furthermore, Joseph (2018) has emphasized that nearly all teachers use 35%–50% of their instructional time questioning students. Within one year, students in one classroom can receive more than 60,000 questions (approximately 12,000 questions yearly have been reported for promoting students’ rational thoughts) (Nappi, 2017). Especially in the 21st century, questioning strategy is essential for stimulating students’ critical thinking skills (Nuryani et al., 2018).

Teachers’ instructional strategy, in this case questioning, aims to stimulate students’ curiosity and maintain their interest by encouraging them to emphasize the content of the lesson, help teachers elucidate their confusion, elicit fundamental structures and vocabularies, check what students understand, and support their learning participation (Yuliawati et al., 2016). However, the questioning strategy does have some weaknesses that must be addressed for optimum effectiveness. Nappi’s study (2017) showed that for teachers to apply the questioning strategy, they need to plan effective questions for developing students’ critical thinking skills. Furthermore, another study (Yuliawati et al., 2016) suggested that the questioning strategy cannot be effective and that students will be unmotivated if teachers’ questioning skill is poor. Additionally, Barjesteh and Moghadam (2014) suggested improving the questioning strategy by allowing students opportunities to question the teacher. Therefore, to understand what strengths or weaknesses occur during instruction, teachers need to reflect on instructional planning, actual classroom implementation, questions’ effects, and the overall educational context.

To ameliorate questioning strategy’s weaknesses, Oo et al. (2021) suggested that the reflective teaching model for reading comprehension (RTMRC) be used to qualify method-centered teaching. In the ELT context, these researchers explained the reflective teaching that is a cyclical process of *planning*, *acting*, *reflecting*, and *evaluating* instruction as it involves the reader, strategy, text, and task. Furthermore, reflective teaching is defined as teachers’ process of attaching meaning to what they do in the classroom (Babaei and Abednia, 2016). It is essential for teachers to evaluate their own teaching critically and use this to improve their effectiveness (Gordon, 2017). Additionally, Valdez et al. (2018) explained that a teacher’s reflective instruction can help improve method-centered teaching effectiveness. The role of reflective teachers is to think, study their instructional process, and focus on the problems or weaknesses in their teaching practices (Wu and Wu, 2016).

In Myanmar, most classroom lessons are teacher-centered. That is, a few decades ago, teachers’ effective questioning and stimulation of critical thinking skills almost disappeared; instead, most students learned through memorization without understanding lessons’ meanings (Soe et al., 2017). Furthermore, many teachers underemphasize lesson preparation and reflection on their instructional processes (Hayden and Martin, 2013). Myanmar’s National Education Strategic Plan (NESP) 2016–2021 encourages teachers to use innovative instructional strategies to match students’ needs and innovative assessment to evaluate their academic achievement (Ministry of Education, 2015). One review report “Strengthening Pre-service Teachers’ Education in Myanmar (STEP)” clearly suggested constructing “a strong and equitable education system in Myanmar that is built around reflective, competent, and qualified teachers” (p. 37). It also recommended that teachers have opportunities to use reflective teaching practices with learner-centered teaching strategies (UNESCO, 2020). These factors inherently call for research based on teachers’ reflective practices within the instructional context. Therefore, we conducted a study based on the reflection-based questioning approach (RBQA) to teach students (English) reading comprehension skills in Myanmar.

## 2 Literature review

### 2.1 Conceptualization of instruction in reading comprehension skills

As a process of meaning-construction based on the reading context, reading is significant in English language learners’ success (Kim et al., 2016). Every day in different ways, we read the news, messages, notes, books, and various other writings. In fact, English language students attain greater achievements if they generally have high reading ability (Puteri et al., 2017). However, students arrive in school from a wide range of backgrounds in family, experiences, and skill in reading comprehension so that teachers struggle to accommodate each student’s needs (Lim et al., 2018). Therefore, comprehension of reading passages is a major skill that provides students with immense information and benefits (Mannong, 2018). Students’ reading comprehension is a process of interrelationship between the readers and the reading text. Considering these presumptions, students with poor reading ability are much less benefited from reading (Lim et al., 2018).

Improving students’ reading comprehension involves a complex interaction between the teachers’ instructional strategy, learning environment, readers’ backgrounds, individual readers, specific tasks, and the text itself (Yang, 2016). In other words, it requires an interactive instructional context involving five factors: strategy, reader, task, text, and context (Suwanto, 2014). For teaching reading text, teachers normally use at least one of the three teaching approaches, such as bottom-up, top-down, and alternate use of bottom-up and top-down (Yang, 2016). Additionally, teachers employ various instructional strategies to help students effectively comprehend reading texts. There were also some studies investigating teachers’ instructional strategies on students’ reading comprehension; for example, Egiyantinah et al.’s (2018) study investigating the effect of the reciprocal teaching strategy and learning styles on students’ reading comprehension achievement; Anyiendah et al.’s (2019) study exploring the effect of the interactive approach on learners’ achievement in reading comprehension in Vihiga, Kenya; Ahmada’s (2019) study examining the effect of the jigsaw learning model in teaching reading comprehension on narrative text; Salari and Hosseini’s (2019) study scrutinizing the effect of the team-based learning strategy on the Iranian intermediate EFL students’ reading comprehension achievement from the Golrizan Language Institute in Mashhad, Iran; and Barua’s (2021) study inquiring into the effect of the paraphrasing strategy on the tertiary level students’ reading comprehension achievement from the Gauhati University, India.

Apart from varied instructional strategies for reading comprehension, teachers should know how to assess students’ reading comprehension, for one, by asking appropriate, carefully planned questions that help students’ progress from one cognitive level to another (Barrett et al., 2017).

### 2.2 Reading comprehension questions

In the instructional process, teachers’ questions are considered as a tool to encourage students to focus on the learning process, pose a challenge for all learners’ responses, provide an opportunity for students’ cognitive process with sufficient wait time, and develop rational thoughts to grasp all opportunities (Yasid et al., 2021). Barjesteh and Moghadam (2014) have also explained that teachers ask questions for two reasons: (1) to stimulate students’ active participation, and (2) to control the progress of teacher–student interaction so as to adjust instructional pace according to students’ understanding. Moreover, Yuliawati et al. (2016) suggested that the questioning strategy helps students develop their interest in the lesson content by promoting students’ rational thoughts, allowing teachers to eliminate their confusing thoughts, highlighting important ideas and concepts, evaluating their understanding, and encouraging their participation in the lesson. Therefore, questions and questioning skills are essential for language teachers to motivate students not only to provide appropriate responses but to ask questions themselves (Bulent et al., 2016).

To go beyond providing students only factual knowledge, teachers should create higher order questions to help students think more deeply (Yasid et al., 2021). Muayanah (2014) noted that teachers’ questions sometimes cause students’ wonder, ambiguity, and confusion; however, well-planned questions stimulate students’ curiosity and active participation in effective discussion.

Maram and Farrah (2019) suggested Barrett’s taxonomy of reading comprehension questions for language teachers to help teachers’ formulate effective questions (Table 1). This taxonomy encompasses five comprehension levels: literal, reorganizational, inferential, evaluative, and appreciative. Specifically, (1) for literal comprehension questions, students directly identify stated information; (2) for reorganizational questions, they order and/or organize presented information in different and meaningful manner; (3) for inferential questions, they respond to information inductively or deductively; (4) for evaluative questions, they make decisions based on stated information; and (5) for appreciative questions, they respond to stated information based on their emotions (Surtantini, 2019).

**Table 1:** Barrett’s taxonomy of reading comprehension levels.

1	**Literal questions** (Recognition or recall of) - details - main ideas - a sequence - comparison - cause and effect relationships - character traits	**Students’ skills** Locate or identify any kind of explicitly stated fact or detail (for example, names of characters or, places, likeness and differences, reasons for actions)	**Examples** - Name the ---. - List the ---. - Identify the ---. - Describe the ---. - Compare the two ---. - Relate the ---.
2	**Reorganizational questions** - classifying - outlining - summarizing - synthesizing	To organize, sort into categories, paraphrase, or consolidate explicitly stated information or ideas in a reading text	- Summarize the main ideas ---. - State the differences ---. - Describe the similarities… - Classify the same ---. - Outline the key ---.
3	**Inferential questions** - main ideas - supporting details - sequence - comparisons - cause and effect relationships - character traits - predicting outcomes - interpreting figurative language	To use conjecture, personal intuition, experience, background knowledge, or clues in a reading text as a basis of forming hypotheses and inferring details or ideas (for example, the significance of a theme, the motivation or nature of a character) that are not explicitly stated in the reading text/material	- Explain the main idea ---. - What is the writer’s intention -? - What do you think ---? - What will be ---? - What will happen ---? - Why is it occurred when ---? - Why did you decide ---?
4	**Evaluative questions** (Judgment of) - reality or fantasy - fact or opinion - adequacy or validity - appropriateness - worth, desirability, and acceptability	To make evaluative judgment (for example, on qualities of accuracy, acceptability, desirability, worth or probability) by comparing information or ideas presented in a reading text using external criteria provided (by other sources/authorities) or internal criteria (students’ own values, experiences, or background knowledge of subject)	- Describe your opinion in detail -. - Do you think that ---? - Discuss critically ---. - Why do you think so ---? - How important is this ---? - What is the moral of the story -? - How is it appropriate with ---? - Why is this purposeful ---?
5	**Appreciative questions** - Emotional response to content - Identification with characters - Reactions to author’s language use - Imagery	To show emotional and aesthetic/literary sensitivity to the reading text and show a reaction to the worth of its psychological and artistic elements (including literary techniques, forms, and styles)	- Discuss your response ---. - Comment on the writer’s use of language ---. - What impression did you get about ---? - Do you like this ---? Why?

*Source*: Adapted from Reeves (2012).

### 2.3 Questioning strategy

Initiated by Socrates more than 2000 years ago, the instructional questioning strategy used here is based on the initiate-response-evaluate (IRE) model, in which the teacher first asks (Initiates) questions related to the text, students then answer (Response), and the teacher finally assesses (Evaluates) responses and/or provides feedback to improve their reading comprehension (Corley and Rauscher, 2013). Questioning strategy encourages teachers to plant seeds of critical thinking in students’ minds (Acim, 2018), so teachers’ questions are crucial in any active instructional process, stimulating students’ reflections and challenging their deep understanding (Yuliawati et al., 2016). However, the teacher should consider questions’ complexity and provide adequate wait time for students to think and then respond (Barrett et al., 2017).

Guihua (2006) suggested the following guidelines to improve teachers’ questioning skills, so that teachers should (1) gain clarity about the question (i.e., issues that the teacher wants the students to know); (2) state the question before calling on an individual student, so that all students can think about it and/or participate in answering; (3) leave adequate time for students to think about and formulate an answer; (4) ask only one question (i.e., asking several questions at a time can confuse students); and (5) ask questions in an easy-to-difficult sequence, so students can actively participate in their learning. In sum, to encourage students’ critical thinking, questions should be clear, precise, relevant, accurate, and sufficiently deep (Elder and Paul, 2007).

### 2.4 Importance of reflection in teaching

Originated by John Dewey (1933), the concept of instructional “reflection” has been employed in education for more than 80 years (Mathieson, 2016). It indicates cyclical behaviors of perceptions and communication analyses with the goal of making teachers’ actions progressive (Kayapinar, 2016). In teaching, “reflection” means the critical thoughts teachers have *before*, *during*, and *after* the instructional process (Edwards, 2017). However, many educators misapprehend reflection as simply thinking about the instructional process, but Mezirow (2006) explained that teachers’ reflective practice is much deeper than simply thinking about teaching experiences. Instead, reflective practices first involve systematic unit and/or lesson planning, next, mindful monitoring of instructional events as they occur, and then evaluating the entire instructional context.

To clarify reflective practice in general, Paterson and Chapman (2013) explained that reflective individuals do not merely think about their past actions but consciously rethink their experiences, actions, and emotions and combine them with their background schema of knowledge to enhance their reading comprehension skills. Furthermore, reflections allow the teacher to become aware of the pros and cons of their instructional process and gain a better understanding of how the teaching method, technique, and materials are proceeding (Töman, 2017). More specifically, for a reflective teacher, the reflection is about their systematic evaluation of teaching practices, with the help of other colleagues’ observation or other reflective tools (Ratminingsih et al., 2017).

Moreover, for teachers to advance their instructional practice, reflective teaching is essential (Mathieson, 2016). Training courses alone cannot prepare teachers to face every classroom challenge. However, at any career stage, teachers can practice reflective teaching, in order to evaluate planning decisions’ appropriateness according to actual instructional events; then, if necessary, they can improve their instruction’s effectiveness (Krulatz, 2016). In other words, reflective teaching is a retrospective method for teachers to explore instructional effectiveness and weakness and to modify the context to create more effective instruction (Afshar and Farahani, 2015; Valdez et al., 2018). Therefore, Oo et al. (2021) suggested the reflective teaching model (involving four stages—*planning*, *acting*, *reflecting*, and *evaluating*) to qualify the instructional context for reading comprehension (involving reader, strategy, text, and task). Therefore, this model was adopted as this study’s conceptual framework.

### 2.5 Conceptual framework

**Figure 1:**
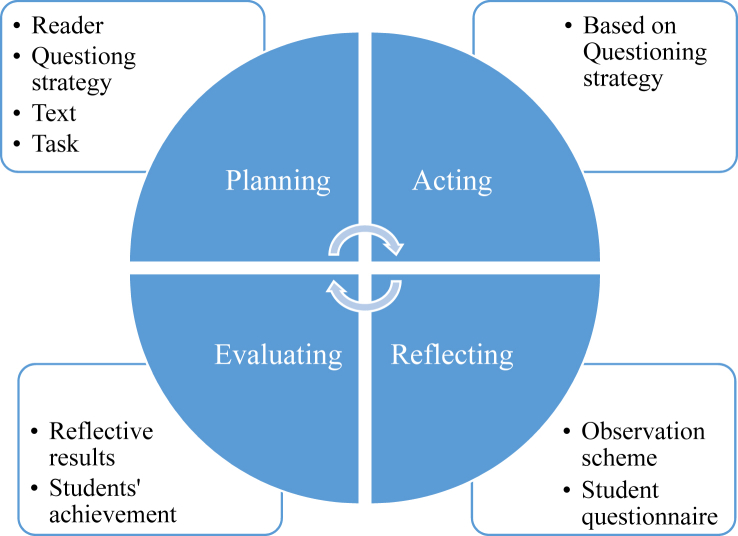
Reflective Teaching Model for Reading Comprehension (RTMRC). *Source***:** Adapted from Oo et al. (2021, p. 4).

In this study, we applied the Reflection-Based Questioning Strategy (RBQA) in which the questioning strategy was utilized within the framework of the Reflective Teaching Model for Reading Comprehension, RTMRC (Oo and Habók, 2020, p. 133; Oo et al., 2021, p. 4), based on teachers’ *planning*, *acting*, *reflecting*, and *evaluating* the instructional context of reader, strategy, text, and task (Figure 1).

In the *planning* stage, the teacher plans how to teach with the questioning strategy based on the IRE model (strategy): the students (reader), reading passages (text), and activities (task) students complete. In the *acting* stage, the teacher instructs students, following the planned questioning strategy procedures above. After a lesson or unit employing this strategy, during the *reflecting* stage, the teacher recalls the instructional context, effect, and outcome, using two reflective tools—the anonymous student questionnaire and the observation scheme suggested by Brookfield (2017). Finally, in the *evaluating* stage, the teacher assesses the instructional context with reflective results and reflective exercises from the text (Oo and Habók, 2020). If weaknesses appear, the teacher can ameliorate them for better results next time. Employing the RBQA as explained above, students are likely to comprehend reading texts well.

### 2.6 Study aim and research questions

Given the framework detailed above, this study aimed to discover aspects of RBQA that affect students’ (English) reading comprehension achievement in Myanmar. The following questions were addressed in this research.

*RQ*_*1*_*: What are the reliability and validity of instruments measuring students’ reading comprehension?*
*RQ*_*2*_*: Is the RBQA instruction effective for students’ reading comprehension?*
*RQ*_*3*_*: What is the effect of teachers’ reflection practices on students’ reading comprehension achievement?*

## 3 Method

There are different types of studies related to the reflection process of teaching–learning situation (e.g., Aliakbari and Adibpour’s [2018] noninterventionist research; Akyıldız and Semerci’s [2016] interventionist research; Wong et al.’s (2009) action research; and Wu and Wu’s (2016) observational research). This study applied a quasi-experimental research method (interventionist research design) to investigate RBQA’s effectiveness on students’ reading comprehension achievement. The study duration was 5 weeks (25 sessions each of 45 min). We selected this (quasi-experimental design) interventionist study because interventionist studies are often representative of natural instructional contexts and they may not differ significantly from what students might perform during their normal classes (Loewen and Philp, 2012). For this study, participants and sampling procedures, instruments, and procedures are described in the following sections.

### 3.1 Participants

Following Sedgwick’s (2014) cluster sampling technique, we first estimated the population as 9th graders (*N* = 1000) of upper secondary schools in Sagaing Township, Myanmar. Second, we chose 10 basic-education upper secondary schools. Using random sampling as the third step, we selected 5 of these 10 schools. Finally, every Grade-9 student (*N* = 458) from these five schools (clusters/groups) participated in the research. From the five schools, English language teachers (*N* = 5) participated by teaching the English text, and their subject deans or colleagues (*N* = 10, i.e., two per each school) joined the study as observers. First, the research was substantially reviewed by the IRB at the Doctoral School of Education, University of Szeged. It was approved that the research was consistent with standardizations recommended by the IRB. Second, the parents and teachers of the participating students were also requested to provide the written consent. Participating schools managed these written consents.

### 3.2 Instruments

#### 3.2.1 Pre- and post-tests

In this study, pre- and post-tests were the main instruments including the same content but different structures. Based on the school textbook for 9th graders prescribed by the Myanmar Ministry of Education, the test questions numbered 23: 8 items for literal comprehension, 2 for reorganizational comprehension, 5 for inferential comprehension, 5 for evaluative comprehension, and 3 for appreciative comprehension. These test items’ construction aligned with Barrett’s taxonomy of reading comprehension (Surtantini, 2019).

#### 3.2.2 Student questionnaire

During the RBQA intervention, teachers reflected the instructional context by using the student questionnaire (students’ feedback) adapted from Richards and Lockhart (2007). Students had to fill the questionnaire based on their learning preferences. Teachers do not fill out the questionnaire; however they reflect on the instructional events based on students’ responses provided in the questionnaire. The questionnaire contains 17 items: 5 for reflection on readers, 5 on strategy, 4 on text, and 3 on task. Previously, we had translated this questionnaire into Burmese and confirmed the translation with four Burmese language experts. It was also validated in a pilot research conducted at a Myanmar upper secondary school with 83 participants a few months ago (Grade 9 students).

The questionnaire had 20 items in pilot testing, but only 17 of them could be used in the main study’s questionnaire (because three items with low factor loadings were suppressed after the pilot study).

#### 3.2.3 Observation scheme

While reflecting on the instructional context, the teacher also used the observation scheme (adapted from Richards and Lockhart, 2007) with the help of 10 observers (two per school) who randomly scrutinized the teachers’ instructional context for approximately 5 times (45 min each). The 10 observers from 5 schools observed the instructional situations of experimental groups for 5 times (total observation = 50 observations).

### 3.3 Procedures

The study included the following four steps. First, a theoretical RTMRC was self-developed, and we face-validated it with four experts (two language specialists and two methodology professionals). The two language specialists were the professors of the English Departments from Sagaing University of Education and Yangon University of Education, Myanmar. The other two methodology professionals were also from the Departments of Methodology and Curriculum Development of Sagaing University of Education and Yangon University of Education, Myanmar. In the second step, we prepared the instruments and validated them with six content experts from Myanmar (three senior English language teachers from upper secondary schools and three English language teachers from the Department of Methodology and Curriculum Development, Sagaing University of Education). Third, we pilot-tested the instruments to confirm their construct validities and then prepared the main research.

The fourth step was conducting the main research by applying the RBQA—that is, employing the questioning strategy within the RTMRC framework. The experimental research with the RBQA teaching uses the same flow of normal teaching–learning situation (following the RBQA teaching for the experimental group but traditional teaching for the control group) (Loewen and Philp, 2012). And we did not deviate their class sessions, teaching hours, and assessment systems. For the RBQA teaching, we provided teachers detailed lesson plans (how to teach experimental students with RBQA). The detailed lesson plans were also easy for them to follow (their actual teaching can slightly deviate with the supported lesson plans based on their teaching experiences, however, we asked them to record such kinds of minor deviations, and combine them with the reflected results for planning next sessions). Therefore, participating teachers from the five selected schools totally agreed to participate in this study. Teachers administered pre-tests to the experimental and control groups before to the RBQA intervention to assess their baseline status. Next, teachers of the experimental group employed RBQA intervention and then reflected on their instructional context, aided by the student questionnaire and the observation scheme. The student questionnaire was used by participating teachers in all sessions of experimental teaching with RBQA. For the case of the observation scheme, observers randomly came to that classroom at least once a week (involving 5 sessions) during experimental teaching (5 total sessions/observations for 25 sessions of experimental teaching with RBQA in this study). Teachers and students were unaware about which sessions would be monitored by the observers. After the RBQA intervention, the two groups completed post-tests to determine RBQA’s effectiveness on students’ reading comprehension achievement.

### 3.4 Data analysis

For measuring the instruments’ internal consistency reliability, we used Cronbach’s Alpha (r); its recommended value is > .6; however, >.7 is better (Gliner et al., 2017). Then, to validate the instruments’ construct validities, we used convergent and discriminant validity measures. The average variance extracted (AVE) (>.5) and the composite reliability, CR (>.7) were also measured for convergent validity; the instruments’ component/factor correlations and the square root of AVE were compared to determine whether the square root of AVE was higher than the factor correlations) for discriminant validities (Habók and Magyar, 2018). We also used Rasch analysis (based on the item response theory) and employed the Quest program to estimate student parameters and item difficulty levels. To investigate RBQA’s effectiveness on students’ reading comprehension, we applied *t-*tests for both independent and paired sample tests (Gliner et al., 2017). According to Goulet-Pelletier and Cousineau (2020), the RBQA’s effect on reading comprehension was quantified by Cohen’s *d* effect size (*d* = .3, small; *d* = .5, medium; *d* = .8 and above, large).

To measure effects of teachers’ reflections on the instructional context, moreover, we employed structural equation modeling (SEM). In the analysis of SEM, standardized root mean square residual (SRMR) was used as the absolute fit index, comparative fit index (CFI) was used to analyze the model’s fit goodness, and root mean square error of estimation (RMSEA) was utilized for the parsimonious fit index (Kline, 2011). The SRMR is acceptable at <.05 (Zhang, 2013), levels of CFI range from 0 to 1 (>.90 is acceptable, and >.95 is good) (Byrne, 2010), and RMSEA values at <.08 (<.05 is acceptable) are good (Habók and Magyar, 2018).

## 4 Findings

*RQ*_*1*_ *: What are the reliability and validity of instruments measuring students’ reading comprehension?*

For addressing RQ_1_, we investigated the instruments’ internal consistency reliability (Cronbach’s Alpha, r) and construct validities (convergent and discriminant validities). Internal consistencies of the overall instruments (pre- and post-tests, r = .76; student questionnaire, r = .72; observation scheme, r = .60) were good and acceptable for use in this study (with the exception of a few component internal consistencies).

For convergent validity measures, almost all of these three instruments’ AVE values were greater than recommended (>.50), and all CR values ranged from .67 to .96, consistent with recommended values; >.70). Accordingly, all instruments’ convergent validities were confirmed (Table 2).

**Table 2:** Instruments’ convergent validity.

Instruments	Factors	No. of Items	Cronbach’s Alpha (>.60)∗	Average Variance Extracted (>.50)∗	Composite Reliability (>.70)∗
Pre- & post-tests	Literal	8	.70	.51	.83
	Reorganizational	2	.45	.51	.85
	Inferential	5	.42	.47	.81
	Evaluative	5	.63	.46	.80
	Appreciative	3	.61	.80	.92
	Total (Overall reliability)	23	.76	.53	.88
Student questionnaire	Reader	5	.70	.81	.90
	Strategy	5	.65	.61	.88
	Text	4	.54	.77	.93
	Task	3	.47	.43	.67
	Total (Overall reliability)	17	.72	.63	.96
Observation scheme	Instructional Process	14	.60	.54	.93

Note: *∗Indicates the acceptable values*.

To inquire about the instruments’ discriminant validities, the interconstruct correlations of the factors/components were compared with the square root of AVE measures. As all values of the square root of the AVE (.678–.894) were greater than all interconstruct values (.020–.228), this study could confirm instruments’ discriminant validities (Table 3).

**Table 3:** Measures of instruments’ discriminant validities.

Instruments	Component Correlation Matrix					
Pre- and post-tests	Components	Literal	Reorganizational	Inferential	Evaluative	Appreciative
	Literal	**.714∗**				
	Reorganizational	.043	**.714∗**			
	Inferential	.191	.160	**.685∗**		
	Evaluative	.142	.228	.064	**.678∗**	
	Appreciative	.147	.175	.092	.020	**.894∗**
Student questionnaire	Components	Reader	Strategy	Text	Task	
	Reader	**.900∗**				
	Strategy	.060	**.781∗**			
	Text	.030	.119	**.877∗**		
	Task	.081	.088	.033	**.655∗**	
Observation scheme	Components	1	2	3	4	
	1	**.800∗**				
	2	.082	**.707∗**			
	3	.083	.136	**.801∗**		
	4	.004	.080	.090	**.591∗**	

Note: *∗Shows the value of square root of AVE*.

Based on internal consistency reliability and construct validities, it was determined that the instruments proved valid to reflect the instructional context and measure students’ performance in reading comprehension.

*RQ*_*2*_*: Is the RBQA instruction effective for students’ reading comprehension?*

*RQ*_*3*_*: What is the effect of teachers’ reflection practices on students’ reading comprehension achievement*

**Figure 2:**
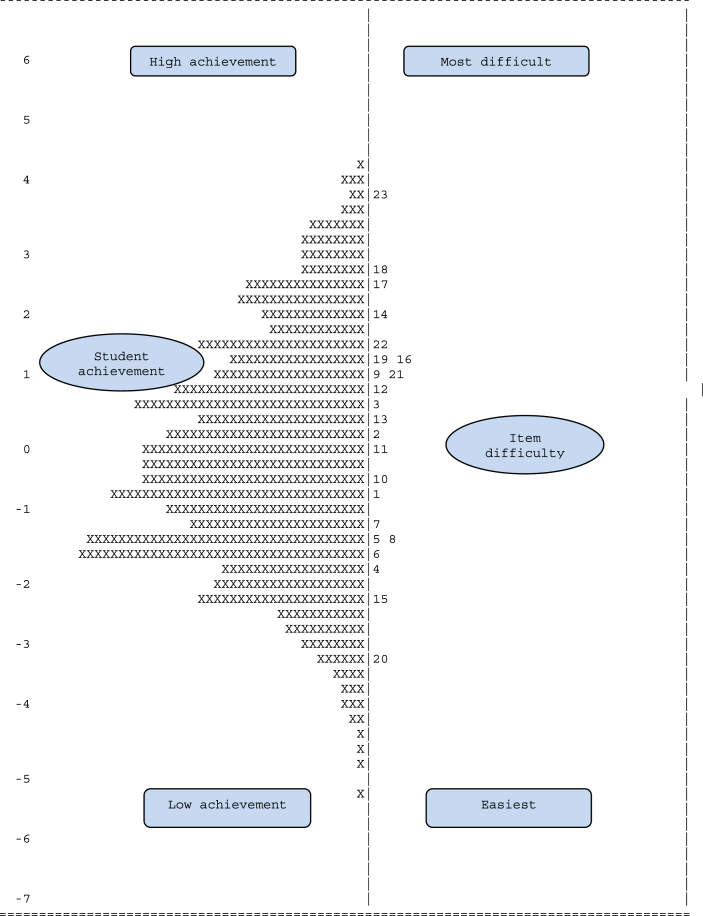
Item–person map of students’ ability and item difficulty levels.

Addressing this question, we compared the achievement of the experimental and control groups. Before investigating RBQA’s effectiveness, we estimated students’ ability parameters and items’ difficulty levels by Rasch analysis and the Quest program. Figure 2 shows the distribution between students’ achievement and difficulty levels of the items.

The left side of Figure 2 displays students’ achievement and the right side indicates items’ difficulty levels. The left’s higher part shows students’ higher achievement and its lower part their lower achievement; the right’s higher part shows more difficult items and its lower part, easier items. Therefore, the graph shows that appreciative (items 17, 18) and reorganizational questions (23) were the most difficult, but evaluative questions (items 15, 20) were the easiest. Even so, most items were at mid-difficulty levels, showing students’ high achievement in literal comprehension (items 6, 7, 8, 9, 10) and inferential comprehension questions (items 1, 2, 3, 4, 5, 12, 14, 22). However, the test’s overall distribution was normal. As for homogeneity, the overall measure of Levene statistic sig-value, *p,* was .073 (Levene statistic sig-value, *p* > .05 recommended by Gliner et al., 2017). Therefore, the entire test was found normal and homogeneous.

Next, we investigated both groups’ initial levels (before RBQA intervention) as shown by pre-test data (maximum score = 45), which were analyzed with the independent samples *t-*test. No significant difference (*p > .5)* appeared between the two groups, indicating nearly the same baseline pre-intervention (M = 13.47, experimental; M = 13.59, control). Table 4 displays these results.

**Table 4:** Results of experimental and control groups’ pre-tests of reading comprehension skills.

Groups	N	M	SD	MD	Effect size (Cohen’s *d*)	*df*	Sig
Experimental	228	13.47	2.106	−.113	0.056 (very low)	456	.572 (n.s)
Control	230	13.59	2.177				

Note: *n.s. = Insignificant*.

After administering the pre-test to both groups, the RBQA intervention was provided to the experimental group but not to the control group. Then, to investigate the RBQA’s effectiveness, we compared the achievement of two groups using data analysis (from post-test scores, maximum score 45 points) and the independent sample *t*-test. There was a significant difference between experimental and control groups (*p* < .001), and the RBQA experimental group’s mean score (M = 31.86) was significantly greater than the control group’s mean score (M 27.04) (Table 5). Therefore, the study results revealed that teaching with RBQA outperformed traditional instruction for reading comprehension.

**Table 5:** Post-test scores of experimental and control groups.

Groups	N	M	SD	MD	Effect size (Cohen’s *d*)	*df*	Sig
Experimental	228	31.86	3.071	4.82	1.25 (high)	456	.000∗∗∗
Control	230	27.04	4.458				

Note: ∗∗∗*p* < 0.001.

Further to investigate RBQA’s effectiveness in teaching reading comprehension, we compared the experimental group’s pre- and post-tests using data analysis and the paired samples *t-*test. Results revealed a significant difference (*p* < .001) between the experimental students’ pre-test (13.47) and post-test (31.86) mean scores. The effect size (Cohen’s *d* = 6.98) of teaching with RBQA between these two tests was extremely large (Table 6). Accordingly, we concluded that the RBQA was effective for students’ reading comprehension.

**Table 6:** Experimental Group’s pre-test and post-test reading comprehension scores.

Experimental group	N	M	SD	MD	Effect size (Cohen’s *d*)	df	Sig
Pre-test	228	13.47	2.106	−18.39	6.98 (very large)	227	.000∗∗∗
Post-test	228	31.86	3.071				

Note: ∗∗∗p < 0.001.

For reflections on instructional context, the teachers used two instruments, the student questionnaire (reflecting students’ eye expressions/opinions) and the observation scheme (reflecting observers’ eyes expressions/opinions). Post-test scores were used as the students’ achievement in reading comprehension. Therefore, to address this question, we investigated relationships between the student questionnaire and students’ achievement, and between the observation scheme and students’ achievement. Using IBM-SPSS Amos 23 software, we employed SEM to investigate the effect of teachers’ reflections on students’ achievement in reading comprehension.

First, in the association model between teachers’ reflections (based on the student questionnaire and the observation scheme) and students’ reading comprehension achievement, no significant difference (*p* > .05) was found. The ratio of Chi-square by degrees of freedom was <3 (*χ*^2^/*df* < 3) (Kline, 2015). Model-fit measures (SRMR = .03, CFI = .97 and RMSEA = .08) were also nearly consistent with recommended values (Table 7). Therefore, the model could be determined as suitable for estimating its related measures.

**Table 7:** Model-fit measures for teachers’ reflections and students’ reading comprehension.

Event	*χ*2	*df*	*p–*value (>.05)	Absolute index, SRMR (<.05)∗	Comparative index, CFI (≥.9)∗	Parsimonious index, RMSEA (<.08)∗
Teachers’ reflection on instructional context	279.54	87	.07	.03	.97	.08

Note: ∗Describes the recommended values: χ2 (chi-square) describes the level of collinearity; SRMR indicates the error amount resulting from evaluation of the specified model; CFI shows the model’s capacity compared with the stage without the model; RMSEA shows the amount of errors residue after the model had been fit.

**Figure 3:**
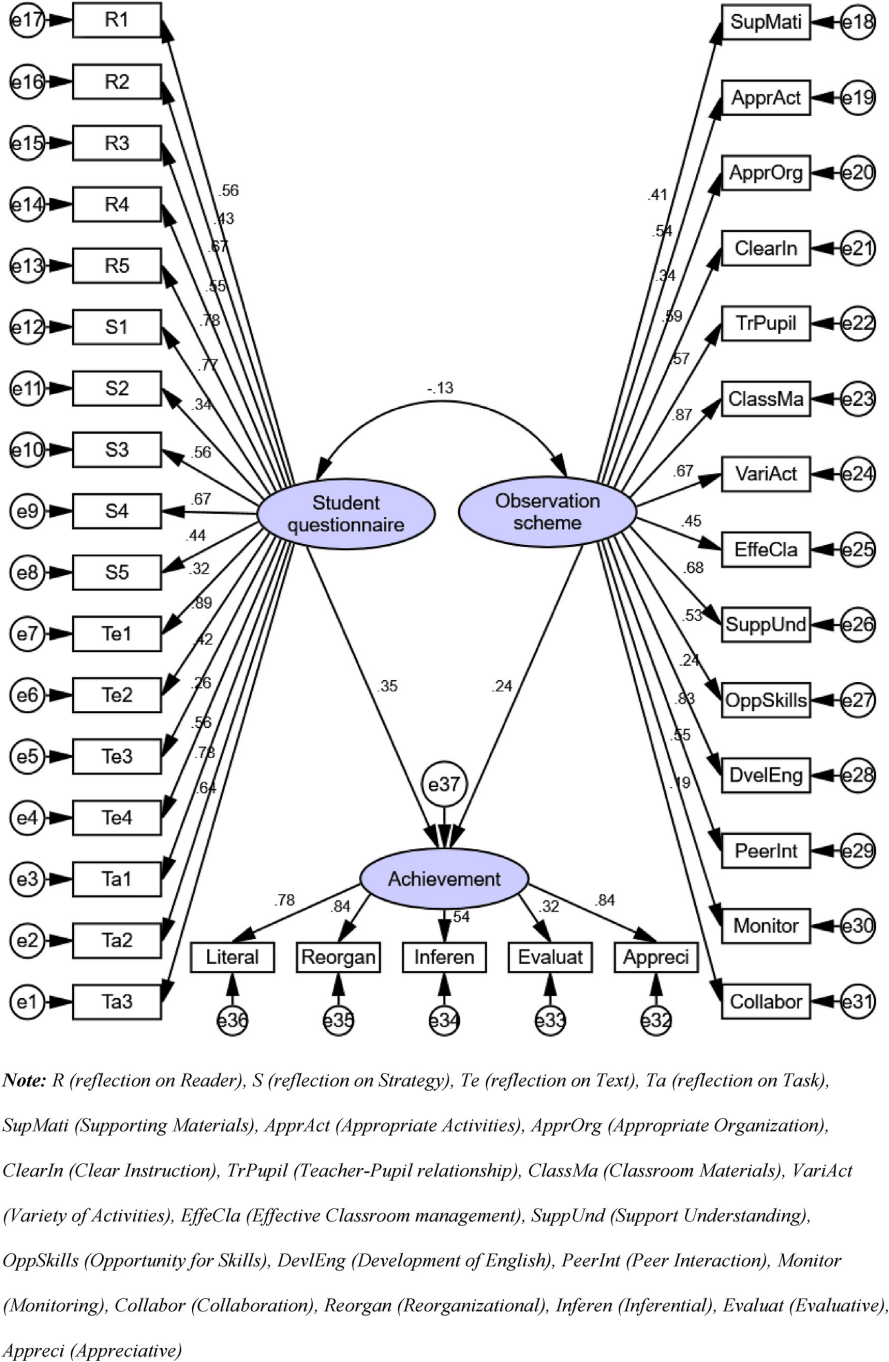
Association Model between Teachers’ Reflection and Students’ Achievement in Reading Comprehension.

The association model (Figure 3) revealed that reflections by both the student questionnaire (*β* = .35, *p* < .05) and the observation scheme (*β* = .24, *p* < .05) had significant, moderately positive impact on students’ achievement (*β* > .4, good; *β* < .4, moderate), as suggested by In’nami and Koizumi (2013). The correlation (r-value) between the student questionnaire and the observation scheme was −.13, not significant (*p* > .05); thus, there was no correlation between the observation scheme and student questionnaire. However, the findings can be construed to show that teachers’ reflective actions on their lesson were significant and had a positive impact on students’ achievement in reading comprehension.

In addressing this research question, teachers’ results were shown to support reflection’s effects on the instructional context. After the RBQA intervention, teachers found both instructional strengths and weaknesses as follows.

### 4.1 Instructional strengths from reflections

Teachers reflected on their instructional contexts with two tools, such as the student questionnaire and observation scheme. Results were considered to be the most frequent responses. Teachers found the following instructional strengths in teaching reading comprehension with RBQA:

• Teachers could create a sensitive classroom environment by interacting with students through stimulus questions.
• Teachers could monitor students’ learning by asking different types of questions.
• The classroom environment was livelier when teachers assigned students peer-group interactions.
• Teachers created better teacher–student relationships.
• By asking questions in English, teachers improved students’ English communication.
• By providing feedback, teachers supported students’ understanding.
• Teachers could manage classroom organization well by asking questions.
• Teachers gave clear instructions and asked clear questions.
• Most students could apply their existing skills and knowledge to answer the teachers’ questions.
• Almost all students appreciated their teachers using the blackboard/whiteboard often while teaching reading comprehension.
• Most students learned better during group work.
• Most students appreciated teachers’ questioning strategy.
• Almost all students mentioned that they could hear their teacher’s voice well.
• Most students responded that for answering teachers’ questions, the text was easy to understand.
• Students mostly enjoyed learning by doing tasks (e.g., taking notes, underlining, highlighting) related to reading texts.
• Students mostly enjoyed teachers’ reading comprehension exercises on reflections.

### 4.2 Instructional weaknesses from reflections (improved in later sessions)

The following are some instructional weaknesses in reading comprehension RBQA instruction:

• Students felt shy when they were asked to do individual tasks (read aloud individually or asked questions individually).
• Students greatly depended on their classmates or teachers (e.g., they wanted the teacher to explain every question).
• Students mostly did not like teachers’ asking more than one question at a time (and wanted to ask teachers some questions).
• When some students asked teachers questions, the teachers did not listen carefully.
• Teachers did not provide adequate wait time for some questions (relatively poorer performing students needed more time to answer).
• When using the questioning strategy, a few teachers failed to provide a variety of activities (e.g., think-pair-share, jigsaw, group discussion).
• A few teachers did not use enough effective teaching aids (e.g., charts, pictures, other technical tools).
• A few teachers needed better classroom management skills when students were assigned group work.
• Based on students’ eye expressions and observers’ suggestions, teachers saw their instructional weak points in the earlier reflections; this qualified them to become better instructors during the later sessions of RBQA teaching. Overall, we saw some improvement in their later instruction (Appendix, Tables 8 and 9).

## 5 Conclusion

Three research questions were addressed in this study. The first research question was about the investigation of reliability and validity of the instruments (pre- and post-tests, student questionnaire, and observation scheme). We could address this research question by confirming their overall construct (convergent and discriminant) validities. However, a few components of instruments revealed low internal consistency reliabilities. But in fact, these three instruments had also been pilot-tested just a few months previously. Thus, these three instruments were appropriate for use in teachers’ reflection on their instruction through RBQA instruction and for measuring Myanmar students’ achievement in reading comprehension.

The second research question concerned RBQA’s effect on Myanmar students’ achievement in reading comprehension. In measuring students’ achievement, we checked the test’s homogeneity and normality measures through Rasch analysis and SPSS Levene statistics. After confirming these measures, we compared students’ pre- and post-test scores (paired samples *t*-test) and the experimental and control groups’ post-test scores (independent samples *t*-test). We also measured the RBQA intervention’s effect size scores. Based on these measures, we concluded that teaching with RBQA significantly impacted students’ achievement in reading comprehension.

The third question concerned the effects of teachers’ reflection (based on students’ and observers’ comments) on students’ performance in reading comprehension tests (post-test scores). To measure the association between teachers’ reflection and students’ achievement, we used IBM-SPSS Amos 23 to perform Rasch analysis, confirming that teachers’ reflections had a significantly positive impact on students’ comprehension of reading passages.

The RBQA combines approaches of Oo et al.’s (2021) RTMRC (based on four steps of *plan, act, reflect,* and *evaluate)* and the questioning strategy (based on the initiate-response-evaluate model). Study results show that this combination approach, RBQA, can immensely benefit both teachers and students during reading comprehension instruction.

During RBQA instruction, students’ (student questionnaire) and observers’ (observation scheme) comments were employed by teachers to reflect on their instructional process. Two example items from the questionnaire were: *“I can actively participate in learning reading comprehension because I hear the English teacher’s voice well*” *and “I like the English teacher’s classroom management.*” However, a few student responses revealed some weaknesses, for example, insufficient teaching aids and poor classroom management. After receiving such feedback from the reflection tools, teachers did improve later instructional sessions. From the observation scheme, teachers also noted some weaknesses: “*lack of different activities*” and “*unclear questioning*,” so in later sessions, they enhanced their questioning strategy.

While teachers reflected on instructional events through reflective tools, students reflected on their learning effectiveness with the help of teachers’ questions related to the reading text. Because students’ higher-level understanding emerges from reflections on learning effectiveness (Mosley Wetzel et al., 2017), RBQA was extremely beneficial and helpful for students’ understanding of the reading text. Apart from this type of reflection, students also had opportunities to express their opinions on teachers’ instructional strategies, learning activities, the reading text, and their own feelings during lessons and learning. In Myanmar culture, students normally refrain from saying “No” when teachers ask, “*Do you understand me?*”; “*Do you like/understand the reading text?*”; “*Do you feel ashamed to read out loud by yourself?*”; *or* “*Do you like this teaching strategy?*” However, in fact, in responding anonymously to the student questionnaire, they clearly expressed likes and dislikes of teachers’ instructional context.

Some studies of questioning strategy that *did not employ* teacher’s reflection recommended certain points to consider. For instance, Nuryani et al. (2018) reported that teachers did not notice students’ eagerness to ask the teacher questions, a failure that could surely cause students to lose interest. Additionally, the teacher should plan various levels of questions; without doing so, questions tend to be at only low or basic levels (Nappi, 2017). Teachers should ask questions but also provide students thinking time, and they should certainly not answer their own questions (Yuliawati et al., 2016). When students respond to questions, teachers should listen attentively, reply positively (e.g., thumbs-up, nodding in agreement, positive comments), and if appropriate, provide feedback (Nuryani et al., 2018). In this study of RBQA teaching, such events and/or weaknesses also occurred in earlier sessions. However, with the help of Oo et al.’s (2021) RTMRC, teachers could diagnose those weaknesses, correct them, and plan better instruction for later sessions.

Like most studies, this one had some limitations that should be corrected in future research: (1) The SEM analysis needed more participants; (2) teacher observations should occur very often (more than five times); and (3) teachers should use more information and communication technology tools in their teaching with RBQA.

In a nutshell, however, this study confirmed that teaching with RBQA profoundly and positively impacted 9th graders’ English reading comprehension achievement from Sagaing Township, Myanmar. It proved that Oo et al.’s reflective teaching model could well employ the questioning strategy in teaching students reading comprehension skills. Therefore, for future research based on this study, we believe that any teaching strategy can be examined and improved by applying the reflective teaching model, a cyclical process of *planning*, *acting*, *reflecting*, and *evaluating*). In the *reflecting* stage, teachers can use various reflective tools, for instance, keeping a diary, tape recording, portfolios, and so on. Such RBQA allows both teacher and students to reflect on the teaching–learning process.

Because of its generalizability to many academic subjects, this approach is invaluable for both teachers and students both in their ELT reading comprehension process and in other academic areas. Because Myanmar’s government is encouraging ELT to promote the national educational education system (Soe, 2015), this paper will be useful for training teachers. Therefore, this classroom-based experimental RBQA research can be a helpful resource, especially for ELT teachers and their students in Myanmar.

## Declarations

### Author contribution statement

Tun Zaw Oo: Conceived and designed the experiments; Performed the experiments; Analyzed and interpreted the data; Contributed reagents, materials, analysis tools or data; Wrote the paper.

Anita Habók: Conceived and designed the experiments; Analyzed and interpreted the data; Contributed reagents, materials, analysis tools or data; Wrote the paper.

### Funding statement

This work was supported by the 10.13039/501100015763University of Szeged Open Access Fund (Grant number: 5619) and by the Research Programme for Public Education Development of the 10.13039/501100003825Hungarian Academy of Sciences [KOZOKT2021-16].

### Data availability statement

The data that has been used is confidential.

### Declaration of interest’s statement

The authors declare no conflict of interest.

### Additional information

No additional information is available for this paper.

## Figures and Tables

**Table 8:** Reflective Results of Student Questionnaire (N = 1,140, five times of reflection)

Reflective events	Levels	1st Reflection (%)	2nd Reflection (%)	3rd Reflection (%)	4th Reflection (%)	5th Reflection (%)
I like the English teacher to explain everything related to the reading tasks.	Strongly Disagree					2.2
	Disagree	.9	.9	.9		.9
	Agree	51.3	46.9	51.8	49.1	54.4
	Strongly agree	47.8	52.2	47.4	50.9	42.5
I feel happy when my English teacher asks me to read the English text out loud alone.	Strongly disagree	1.8	2.6	.9		
	Disagree	7.0	7.5	4.8	4.8	
	Agree	44.7	40.4	52.6	59.6	46.9
	Strongly agree	46.5	49.6	41.7	35.5	53.1
I like the English teacher to use the blackboard/chalkboard while teaching reading comprehension.	Strongly disagree			2.6	.9	
	Disagree	.9	.9	5.7	2.2	4.4
	Agree	51.3	46.9	56.6	50.9	56.6
	Strongly agree	47.8	52.2	35.1	46.1	39.0
When I don’t understand something while reading the English text, I like to guess the meaning by connecting with other related words.	Strongly disagree	7.9	2.6	1.3		1.8
	Disagree	18.9	9.2	3.9	1.3	3.9
	Agree	48.7	44.3	51.3	61.0	50.4
	Strongly agree	24.6	43.9	43.4	37.7	43.9
I do better at reading in English when I work with others.	Strongly disagree			.4		.4
	Disagree	.9	.9	1.3	1.3	2.6
	Agree	51.3	46.9	56.1	56.6	53.1
	Strongly agree	47.8	52.2	42.1	42.1	43.9
I like the reading techniques the English teacher uses because they help me remember the vocabulary.	Strongly disagree	.4	2.6	3.1	2.6	.4
	Disagree	.4	13.6	10.1	16.7	11.4
	Agree	34.6	47.8	55.3	58.3	53.9
	Strongly agree	64.5	36.0	31.6	22.4	34.2
I like the English teacher using the relevant questions while teaching the reading text.	Strongly disagree			.4		.9
	Disagree	.9	.9	4.8	1.8	3.5
	Agree	51.3	46.9	58.8	57.9	52.2
	Strongly agree	47.8	52.2	36.0	40.4	43.4
I like the strategy the English teacher uses in teaching the reading passages.	Strongly disagree	2.2	2.6	.9	.4	
	Disagree	11.4	10.1	2.6	7.0	1.8
	Agree	48.2	59.6	58.8	60.1	61.0
	Strongly agree	38.2	27.6	37.7	32.5	37.3
I like the English teacher’s classroom management.	Strongly disagree	2.6	.4	.4		
	Disagree	8.3	1.8	3.5	.9	.9
	Agree	52.6	54.8	53.9	50.9	59.6
	Strongly agree	36.4	43.0	42.1	48.2	39.5
I can actively participate in learning reading comprehension because I hear the English teacher’s voice well.	Strongly disagree	1.3	.9	1.3	.4	1.3
	Disagree	5.3	4.8	9.2	6.1	4.4
	Agree	47.4	51.8	57.5	57.5	57.0
	Strongly agree	46.1	42.5	32.0	36.0	37.3
I like the reading text because it is very interesting when the teacher provides us with the reflective questions.	Strongly disagree				.4	.4
	Disagree	.9	.9	.9	1.3	1.3
	Agree	59.6	50.9	56.1	56.6	52.6
	Strongly agree	39.5	48.2	43.0	41.7	45.6
	Total	100.0	100.0	100.0	100.0	100.0
I like the reading text because it is easy to take out the questions from the reading passages to discuss.	Strongly disagree	5.7	3.1	1.8	1.3	
	Disagree	12.3	14.5	10.5	7.0	7.5
	Agree	47.4	53.1	52.6	57.0	61.8
	Strongly agree	34.6	29.4	35.1	34.6	30.7
I like the reading text because it is easy to catch the main ideas to summarize it.	Strongly disagree	.9	.4	.4	2.2	1.3
	Disagree	7.0	4.8	3.9	5.7	4.8
	Agree	53.5	48.2	53.5	46.1	54.8
	Strongly agree	38.6	46.5	42.1	46.1	39.0
The reading text looks difficult to understand; however, I like it because it is easy to answer reading comprehension questions after the teacher’s explanation.	Strongly disagree	1.3	2.6	6.6		
	Disagree	13.6	4.8	15.8	7.0	2.6
	Agree	53.9	50.9	49.1	56.1	63.6
	Strongly agree	31.1	41.7	28.5	36.8	33.8
I like learning by doing tasks (e.g., taking notes, underlining, highlighting) related to reading texts.	Strongly disagree	14.0	3.1	6.1	1.3	.4
	Disagree	26.3	11.0	18.0	14.5	11.8
	Agree	39.0	53.5	55.3	49.1	52.6
	Strongly agree	20.6	32.5	20.6	35.1	35.1
I like to participate in the collaborative activities of learning reading comprehension.	Strongly disagree		.4			.4
	Disagree	2.6	4.8	2.6	1.8	3.5
	Agree	51.8	47.8	49.1	46.5	51.3
	Strongly agree	45.6	46.9	48.2	51.8	44.7
I like the teacher giving us various types of reading comprehension exercises.	Strongly disagree		3.1	5.3	.4	
	Disagree	18.0	11.8	18.0	11.8	7.5
	Agree	52.2	55.3	53.1	52.2	53.5
	Strongly agree	29.8	29.8	23.7	35.5	39.0

**Table 9:** Reflective Results by Observation Scheme (N = 50, five times of reflection)

Reflective events	Levels	1st Reflection (%)	2nd Reflection (%)	3rd Reflection (%)	4th Reflection (%)	5th Reflection (%)
The appropriateness of the selection of materials	Very poor		10.0			
	Poor	10.0	30.0		20.0	30.0
	Good	90.0	60.0	80.0	80.0	70.0
	Excellent			20.0		
The appropriateness of planning the activities	Very poor	20.0				
	Poor	60.0	90.0	40.0		
	Good	20.0	10.0	60.0	40.0	80.0
	Excellent				60.0	20.0
The appropriateness of the organization of the class	Very poor					
	Poor					
	Good	70.0	50.0	30.0	60.0	20.0
	Excellent	30.0	50.0	70.0	40.0	80.0
Clear instructions and models of English language use	Very poor					
	Poor	30.0	20.0			
	Good	70.0	70.0	80.0	70.0	70.0
	Excellent		10.0	20.0	30.0	30.0
Effective teacher/pupil interaction	Very poor					
	Poor					
	Good	80.0	70.0	70.0	100.0	70.0
	Excellent	20.0	30.0	30.0		30.0
Effective organization and management of the whole class	Very poor	10.0				
	Poor	40.0	30.0	10.0		
	Good	50.0	70.0	70.0	40.0	70.0
	Excellent			20.0	60.0	30.0
A variety of activities	Very poor	20.0				
	Poor	80.0				
	Good		80.0	70.0	100.0	90.0
	Excellent		20.0	30.0		10.0
Effective materials	Very poor	30.0				
	Poor	70.0	20.0	20.0	10.0	
	Good		80.0	80.0	50.0	100.0
	Excellent				40.0	
Support for understanding	Very poor					
	Poor	30.0	20.0	40.0	30.0	20.0
	Good	50.0	70.0	60.0	70.0	60.0
	Excellent	20.0	10.0			20.0
Opportunities for learners to apply their existing skills and knowledge	Very poor					
	Poor			10.0		
	Good	70.0	70.0	70.0	70.0	60.0
	Excellent	30.0	30.0	20.0	30.0	40.0
Opportunities for developing English language use	Very poor					
	Poor				10.0	
	Good	80.0	70.0	50.0	60.0	70.0
	Excellent	20.0	30.0	50.0	30.0	30.0
Opportunities for peer-group interaction	Very poor					
	Poor	40.0	30.0	10.0		
	Good	40.0	40.0	30.0	50.0	50.0
	Excellent	20.0	30.0	60.0	50.0	50.0
Effective monitoring of learning	Very poor					
	Poor	10.0	20.0	10.0		10.0
	Good	90.0	70.0	80.0	90.0	80.0
	Excellent		10.0	10.0	10.0	10.0
A sensitive environment for individual learners and their communicative needs	Very poor					
	Poor	20.0				
	Good	40.0	30.0	40.0	80.0	50.0
	Excellent	40.0	70.0	60.0	20.0	50.0

## Appendix A Supplementary data

Student questionnaire 2022 01 07 Student questionnaire 2022 01 07
