# Supplementary material for: Reflection-based questioning: Aspects affecting Myanmar students’ reading comprehension
Source: Heliyon. 2022 Jul 4;8(7):e09864. doi: 10.1016/j.heliyon.2022.e09864 (PMC9260630; doi:10.1016/j.heliyon.2022.e09864)
Supplement: Student questionnaire 2022 01 07 [file mmc1.docx]

**Student Questionnaire**

**Student Gender: ___________________ Date: _______/_______/_________**

**School Name: ______________________ Teacher Name: ________________**

*For each statement, please tick (✓) on the number that best fits your opinion.*

| **Statements** | **Strongly disagree** | **Disagree** | **Agree** | **Strongly agree** |
| --- | --- | --- | --- | --- |
| 1. I like the English teacher to explain everything related to the reading tasks. | 1 | 2 | 3 | 4 |
| 1. I feel happy when my English teacher asks me to read the English text out loud alone. | 1 | 2 | 3 | 4 |
| 1. I like the English teacher to use the blackboard/chalkboard while teaching reading comprehension. | 1 | 2 | 3 | 4 |
| 1. When I don’t understand something while reading the English text, I like to guess the meaning by connecting with other related words. | 1 | 2 | 3 | 4 |
| 1. I do better at reading in English when I work with others. | 1 | 2 | 3 | 4 |
| 1. I like the reading techniques the English teacher uses because they help me remember the vocabulary. | 1 | 2 | 3 | 4 |
| 1. I like the English teacher using the relevant questions while teaching the reading text. | 1 | 2 | 3 | 4 |
| 1. I like the strategy the English teacher uses in teaching the reading passages. | 1 | 2 | 3 | 4 |
| 1. I like the English teacher’s good classroom management. | 1 | 2 | 3 | 4 |
| 1. I can actively participate in learning reading comprehension because I hear the English teacher’s voice well. | 1 | 2 | 3 | 4 |
| 1. I like the reading text because it is very interesting when the teacher provides us with the reflective questions. | 1 | 2 | 3 | 4 |
| 1. I like the reading text because it is easy to take out the questions from the reading passages to discuss. | 1 | 2 | 3 | 4 |
| 1. I like the reading text because it is easy to catch the main ideas to summarize it. | 1 | 2 | 3 | 4 |
| 1. The reading text looks difficult to understand; however, I like it because it is easy to answer reading comprehension questions after the teacher’s explanation. | 1 | 2 | 3 | 4 |
| 1. I like learning by doing tasks (e.g., taking notes, underlining, highlighting) related to reading texts. | 1 | 2 | 3 | 4 |
| 1. I like to participate in the collaborative activities of learning reading comprehension. | 1 | 2 | 3 | 4 |
| 1. I like the teacher giving us various types of reading comprehension exercises. | 1 | 2 | 3 | 4 |
